# Estrogen Receptor, Inflammatory, and FOXO Transcription Factors Regulate Expression of Myasthenia Gravis-Associated Circulating microRNAs

**DOI:** 10.3389/fimmu.2020.00151

**Published:** 2020-02-21

**Authors:** Alyson A. Fiorillo, Christopher R. Heier, Yu-Fang Huang, Christopher B. Tully, Tanel Punga, Anna Rostedt Punga

**Affiliations:** ^1^Center for Genetic Medicine Research, Children's Research Institute, Washington, DC, United States; ^2^Genomics & Precision Medicine, The George Washington University, Washington, DC, United States; ^3^Department of Neuroscience, Clinical Neurophysiology, Uppsala University, Uppsala, Sweden; ^4^Department of Medical Biochemistry and Microbiology, Uppsala University, Uppsala, Sweden

**Keywords:** microRNA, myasthenia gravis, estradiol, miR-21-5p, NF-κB, FOXO

## Abstract

MicroRNAs (miRNAs) are small non-coding RNA molecules that regulate important intracellular biological processes. In myasthenia gravis (MG), a disease-specific pattern of elevated circulating miRNAs has been found, and proposed as potential biomarkers. These elevated miRNAs include miR-150-5p, miR-21-5p, and miR-30e-5p in acetylcholine receptor antibody seropositive (AChR+) MG and miR-151a-3p, miR-423-5p, let-7a-5p, and let-7f-5p in muscle-specific tyrosine kinase antibody seropositive (MuSK+) MG. In this study, we examined the regulation of each of these miRNAs using chromatin immunoprecipitation sequencing (ChIP-seq) data from the Encyclopedia of DNA Elements (ENCODE) to gain insight into the transcription factor pathways that drive their expression in MG. Our aim was to look at the transcription factors that regulate miRNAs and then validate some of those *in vivo* with cell lines that have sufficient expression of these transcription factors This analysis revealed several transcription factor families that regulate MG-specific miRNAs including the Forkhead box or the FOXO proteins (FoxA1, FoxA2, FoxM1, FoxP2), AP-1, interferon regulatory factors (IRF1, IRF3, IRF4), and signal transducer and activator of transcription proteins (Stat1, Stat3, Stat5a). We also found binding sites for nuclear factor of activated T-cells (NFATC1), nuclear factor kappa-light-chain-enhancer of activated B cells (NF-κB), early growth response factor (EGR1), and the estrogen receptor 1 (ESR1). AChR+ MG miRNAs showed a stronger overall regulation by the FOXO transcription factors, and of this group, miR-21-5p, let-7a, and let 7f were found to possess ESR1 binding sites. Using a murine macrophage cell line, we found activation of NF-κB -mediated inflammation by LPS induced expression of miR-21-5p, miR-30e-5p, miR-423-5p, let-7a, and let-7f. Pre-treatment of cells with the anti-inflammatory drugs prednisone or deflazacort attenuated induction of inflammation-induced miRNAs. Interestingly, the activation of inflammation induced packaging of the AChR+-specific miRNAs miR-21-5p and miR-30e-5p into exosomes, suggesting a possible mechanism for the elevation of these miRNAs in MG patient serum. In conclusion, our study summarizes the regulatory transcription factors that drive expression of AChR+ and MuSK+ MG-associated miRNAs. Our findings of elevated miR-21-5p and miR-30e-5p expression in immune cells upon inflammatory stimulation and the suppressive effect of corticosteroids strengthens the putative role of these miRNAs in the MG autoimmune response.

## Introduction

MicroRNAs (miRNAs) are small non-coding RNA molecules that regulate important intracellular biological processes. The field of circulating miRNAs in autoimmune disorders, especially as potential biomarkers have expanded in the recent years, including myasthenia gravis (MG) (1). In MG there is a shortage of biomarkers to predict the clinical course and this is particularly troublesome due to the fluctuating nature of skeletal muscle fatigue with periods of unpredictable worsening (2). MG is a heterogeneous disease that still can be subdivided into two main subgroups based on antibody target: patients with antibodies against the acetylcholine receptors (AChR+ MG; ~85%) and those with antibodies against the muscle-specific tyrosine kinase (MuSK+ MG; ~7%) (3). These two antibody subtypes differ, for example, in the involvement of the thymus which is considered to play a major role in AChR+ MG, especially in terms of thymus inflammation (hyperplasia) but also in regards to treatment response (4).

Recent reports have defined different signatures of elevated serum miRNAs in AChR+ and MuSK+ MG (1). In AChR+ MG, the miRNAs miR-150-5p, and miR-21-5p were upregulated compared to healthy controls and these levels were also lower in patients treated with immunosuppression as compared to immunosuppression naïve patients (5, 6). In addition to miR-150-5p and miR-21-5p, levels of miR-30e-5p were found to be upregulated in late onset AChR+ MG patients and expression of these miRNAs correlated with disease course (7). Interestingly, miR-30e-5p levels were found to be higher in patients with ocular AChR+ MG who later develop generalized MG as compared to those that remain ocular. Given this, expression of miR-30e-5p has the potential to predict clinical disease course (8). On the other hand, the miRNA profile in sera from MuSK+ MG patients revealed elevated miR-151a-3p, let-7a-5p, let-7f-5p, and miR-423-5p levels (9).

miRNAs modulate gene expression by targeting multiple mRNAs encoding proteins involved in a variety of signaling pathways and cellular processes (10). In a similar manner, miRNAs could influence and alter the autoimmunity signaling pathway involved in autoimmune disorders such as MG. Several pathways including the Nuclear factor kappa-light chain-enhancer of activated B cells (NF-κB) pathway are inappropriately regulated in several inflammatory disorders including chronic muscle inflammation (11). Toll-like receptor signaling activates NF-κB-driven inflammation. Elevated levels of Toll-like receptors (TLRs) 2, 3, 4, 5, 8, and 9 have been detected in MG peripheral blood mononuclear cells (PBMCs) (12). Moreover, TLR4, TLR7, and TLR9 have been implicated in amplifying autoimmunity in the MG thymus (13, 14).

In MG, T regulatory cells (Tregs) from the thymus of MG patients are profoundly defective in their suppressive activity (15) and there is also an imbalance in peripheral blood Tregs (16). Therefore, genes involved in these two pathways, Treg differentiation and NF-κB signaling, are anticipated to be associated with MG predisposition. Nevertheless, it is not clear which transcription factors regulate the MG-associated miRNA expression in AChR+ vs. MuSK+ MG. In this study, we examined the regulatory transcription factors that drive expression of extracellular miRNAs found in the serum of patients with AChR+ and MuSK+ MG. Our overall objective in this analysis was to outline transcription factors that regulate these miRNAs and then validate a subset of these (i.e., NF-κB and Estrogen Receptor, ESR1) *in vivo* with cell lines that have sufficient expression of these transcription factors [RAW macrophage cells for NF-κB (17) and T-cells for ESR (18)].

## Materials and Methods

### Bioinformatics

We examined the surrounding regulatory region of each miRNA gene to gain insight into the mechanisms of response to treatment as previously reported (19). Briefly, we examined the binding of transcription factors that are most relevant in MG (20–22) using chromatin immunoprecipitation sequencing (ChIP-seq) data. ChIP-seq data from the Encyclopedia of DNA Elements (ENCODE) was queried for physical binding to DNA loci encoding the human miRNA target of interest (23, 24). Both the independent promoter/enhancer of the miRNA was queried, and for miRNAs that were encoded within introns of a gene, the promoter and enhancer of that gene was additionally queried. The proximal promoter was considered the region directly upstream of the miRNA or gene, within 2 kb (25) while the enhancer was considered the region within 10 kb of the miRNA or gene (26). In addition, we examined the following histone modifications which are enriched at regulatory elements such as promoters or enhancers: histone H3K4 trimethylation (found near promoters), H3K4 monomethylation (found near regulatory elements), and H3K27 acetylation (found near active regulatory elements). For each of these analyses, we used UC Santa Cruz (UCSC) Genome Browser Release 4 (https://genome.ucsc.edu/index.html) with alignment to the GRCh37/hg19 genome build. Each ChIP-seq dataset was analyzed using the ENCODE Regulation Super-Track listed under the Regulation menu. Transcription factors were assayed using the Txn Factor ChIP Track. In regions bound by each transcription factor, DNA motifs recognized by that transcription factor were identified through the Factorbook repository within this track. Consensus motif sequence logo pictograms for each transcription factor were also visualized through Factorbook. Histone modifications were examined using the Layered H3K4Me1, Layered H3K4Me3, and Layered H3K27Ac Tracks. Raw data images for visualization of gene loci and ChIP-seq data were obtained using the PDF/PS function in the View menu of the genome browser. Binding of transcription factors was queried in ChIP-seq datasets produced using all 9 cell line tracks to identify all possible transcription factor binding. These include: GM12878, a lymphoblast cell line; H1-hESC, an embryonic stem cell line; HeLa-S3, a cervical cancer cell line; HepG2, a liver cancer cell line; HSMM, a human skeletal muscle myoblast cell line; HUVEC, a human umbilical vein endothelial cell line; K562, a human immortalized myelogenous leukemia cell line; NHEK, Human Epidermal Keratinocytes; and NHLF; a human lung fibroblast cell line. Histone modifications were also queried in ChIP-seq datasets using all nine available cell line tracks.

### Circulating miR-21-5p Analysis in Patients and Healthy Controls

Detailed patient characteristics have been described previously (5). In brief, stored serum samples were collected in tubes without additives from 54 healthy blood donors (27 females, mean age: 45 ± 16 years; 27 male, mean age: 47 ± 15 years) without any medications and from 73 MG patients (42 females; mean age: 57 ± 15 years), at Uppsala University Hospital. The MG group consisted of 27 female MG patients without any immunosuppression (mean age: 55 ± 13 years), 10 male MG patient without any immunosuppression (mean age: 62 ± 18 years), 15 female MG patients with immunosuppression (mean age: 53 ± 15 years), and 21 male MG patients with immunosuppression (mean age: 62 ± 14 years). In the MG patient cohort without immunosuppression, 76% were AChR+ and 24% were AChR/MuSK antibody seronegative (AChR/MuSK-) and among those with immunosuppression 70% were AChR+ and 30% were AChR-/MuSK-. Mean MGC score was 6.1 (range: 0–34) in the group without immunosuppression and 4.6 (range: 0–18) in the group with immunosuppressive treatment (5). The study was approved by the local Ethical Review Board in Uppsala (Dnr 2010/446) and all patients signed informed consent. RNA isolation, cDNA synthesis and qPCR were performed as previously described (5, 6). In brief, total RNA was isolated from 200 μl serum by using miRCURY™ RNA Isolation Kit—Biofluids (Exiqon#300112) and 2 μl of isolated RNA sample was applied for cDNA synthesis in a 10 μl reaction mix by using Universal cDNA Synthesis Kit II (Exiqon #20330). The RT-qPCR analysis was performed with ExiLENT SYBR® Green master mix (Exiqon #203421) and cDNA reactions were diluted 100X. The following quality controls were included on Pick-&-Mix microRNA PCR panel plates: the interpolate calibration (UniSp3), RNA extraction control (UniSp2 and UniSp4), cDNA synthesis control (UniSp6), and hemolysis test (miR- 23a-3p–miR-451a) (Exiqon). The ΔCT value of hemolysis markers [ΔCT (hemolysis) = CT(miR-23a-3p) – CT(miR-451a)], was used to detect hemolysis and samples with a ΔCT >7 were excluded. Reference miRNAs were miR-93-5p, miR-191-5p, miR-423-4p, and miR-103a and quantification of relative miRNA expression was performed with the comparative CT method.

### ELISA

Serum samples from 20 female AChR+ MG patients (mean age: 54 ± 12 years) without immunosuppressive medication that were also analyzed for miR-21-5p levels, and 20 age matched female healthy controls (mean age: 51 ± 14 years) were analyzed. Enzyme-linked immunosorbent assay (ELISA) was performed for 17-β-estradiol using Human Estradiol kit (KAQ0621, Invitrogen), following the manufacturer's instructions. All samples were assayed in duplicate at an absorbance of 450 nm, and the detection range was 13–935 pg/ml. Inter-assay CV was 6.1%. Calculating and analysis of data was conducted by Spark® Multimode Microplate Reader (Tecan Trading AG).

### miR-21-5p Expression in T Cells Treated With β-Estradiol and 4-Hydroxytamoxifen

Human T cell line KE-37 (DSMZ, ACC 46) was used for β-estradiol (E2758, Sigma) and 4-hydroxytamoxifen (H7904, Sigma) treatments, since human T cells express estrogen receptor (18).

Cells were plated in 35 mm dishes with RPMI1640 media (R7509, Sigma) with 10% charcoal stripped FBS (TA3382101, Thermo Fisher Scientific) at a density of 1,000,000 cells/dish. Each condition was performed in triplicate.

For β-estradiol and 4-hydroxytamoxifen co-treated group, cells were treated with 0.1 nM β-estradiol and 10^−^μM 4-hydroxytamoxifen for 24 h. For dose-response and time-course group, cells were treated for 24, 48, and 72 h, respectively, with ß-estradiol at 0, 5, and 50 nM. The expression of miR-21-5p was examined and analyzed by qRT-PCR, using reference genes miR-103a and miR-191-4p for normalization. miRNA isolation was performed using miRNeasy Mini Kit (217004, Qiagen) and cDNA was synthesized. PCR amplification system included cDNA templates (1:50 dilution), ExiLENT SYBR® Green Master Mix (201420-01, Exiqon) and miRNA-specific primers (Qiagen). PCR amplification was conducted in presence of Rox Reference Dye by Applied Biosystems 7900HT Fast Real-Time PCR system (Life Technologies). Log conversion of the miRNA expression data was done in order to obtain data more similar to a normal distribution for the statistical tests.

### miRNA-Induction in Macrophage Cells

The murine macrophage cell line RAW 264.7, a standard model system for assessment of LPS-induced inflammatory response (17), was cultured in DMEM with 10% FBS. Cells were seeded in a 6-well plate at a density of 500,000 cells/well. Each condition was performed in triplicate. Cells were pretreated with 10 μM of the indicated drug or a DMSO vehicle-only control. Inflammatory signaling was induced using LPS (Thermo Fisher Scientific) or a PBS-only vehicle control at a dilution of 1:1,000 as previously reported (27). After 48 h exosomes were collected from the media using ExoQuick (SBI), and RNA was subsequently extracted using TRIzol. Cells were also lysed for RNA using TRIzol at 24 or 48 h post-LPS induction. Expression of exosome-specific miRNAs was quantified by TaqMan Assay and results were normalized to the Geometric Mean of miR-17 and miR-93 as these miRNAs are reported to remain unchanged in exosomes (28). Expression of cell-specific miRNAs was also quantified by TaqMan Assay and values were normalized to sno202. The Taqman IDs for all miRNAs queried is as follows: snoRNA202, 001090; miR-93, 001090; miR-17, 002308; miR-151-3p, 001190; miR-21-5p, 000397; let-7a, 000377; let-7f, 000382, miR-150−5p, 000473; miR-30e-5p, 002223; miR-423-5p, 002340.

### Statistical Analysis

D Ágostino & Pearson normality test revealed that qPCR data were normally distributed (p>0.05) whereas ELISA data were not (*p* < 0.05). Comparison of miR-21-5p serum data between male and female controls and MG patients was done using one-way ANOVA, followed by unpaired *T*-test comparing female controls and MG patients as well as male controls and MG patients. Comparison of 17-β-estradiol levels between female MG patients and healthy controls was done by Wilcoxon matched-pairs signed rank test. A *p* < 0.05 was considered significant. Spearman Rank correlation was used to determine any correlation between estradiol and miR-21-5p levels as well as between miR-21-5p and age.

## Results

### Transcription Factors Regulating AChR+ vs. MuSK+ MG-Associated miRNAs

**Figure 1:**
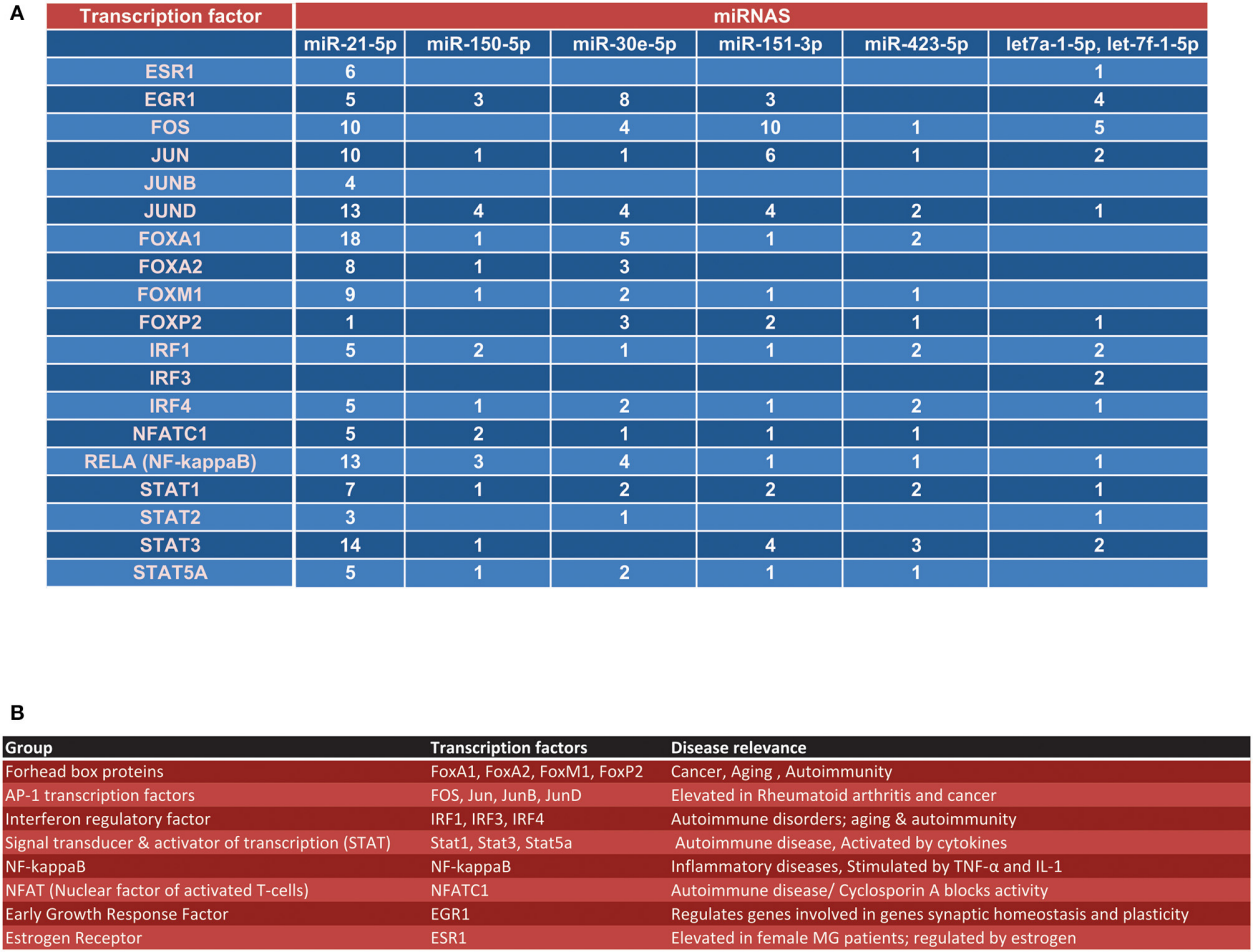
Overview transcription factors that regulate MG-associated miRNAs. **(A)** Using the UCSC Genome browser, each of the seven miRNAs identified from our previous reports (1, 5) were queried for transcription factor regulation. **(A)** Shows an overview of all transcription factor binding sites within the proximal promoter (~2 kb) of the miRNA, the proximal promoter of the gene surrounding the miRNA locus (if applicable), the enhancer region (within ~10 kb) of the miRNA, and the enhancer region of the gene surrounding the miRNA locus (if applicable). **(B)** Overview of the disease relevance and function for each major transcription factor group listed in **(A)**.

The previously identified MG-associated miRNAs in serum included miR-150-5p, miR-21-5p and miR-30e-5p (AChR+ MG) as well as miR-151, miR-423, let-7f, and let-7a (MuSK+ MG) (1). Our bioinformatics analysis of the ChIP-seq data from ENCODE revealed several transcription factor binding sites on the aforementioned miRNA genes (Figure 1). For this analysis, we utilized data from all available cell lines deposited into ENCODE, to get an overview of the regulation of each miRNA. We used an arbitrary cutoff of 2 kb for the proximal promoter region and 10 kb for the proximal enhancer region. To increase confidence that the regions analyzed were bona fide regulatory elements we also overlayed ChIP-seq data showing verified histone modifications indicative of active promoters (H3K4 trimethylation) and enhancers/regulatory regions (H3K4 monomethylation, H3K27 acetylation (Figure 1).

At least nine transcription factors were found to have binding sites in the defined regulatory regions of 7 MG-associated miRNAs. The identified transcription factors included the Forkhead box proteins (FoxA1, FoxP2), the interferon regulatory factors (IRF1, IRF4), signal transducer and activator of transcription proteins (STAT1), AP-1 transcription factors (FOS, Jun, JunB) and NF-κB (denoted as RELA in ENCODE) (Figure 1). When we looked at the totality of transcription factor binding sites on the 7 miRNA regulator regions queried, we found that FOS, JunD, FoxA1, and RELA had the highest occupancy on the MG-associated miRNAs (*N* = 30, 28, 27, and 23, respectively). Intriguingly, only miR-21-5p, let-7a, and let-7f were found to possess estrogen receptor 1 (ESR1) binding sites. We also found nuclear factor of activated T-cells (NFATC1) and early growth response factor (EGR1) binding sites on the majority of the miRNA genes queried (Figure 1).

**Figure 2:**
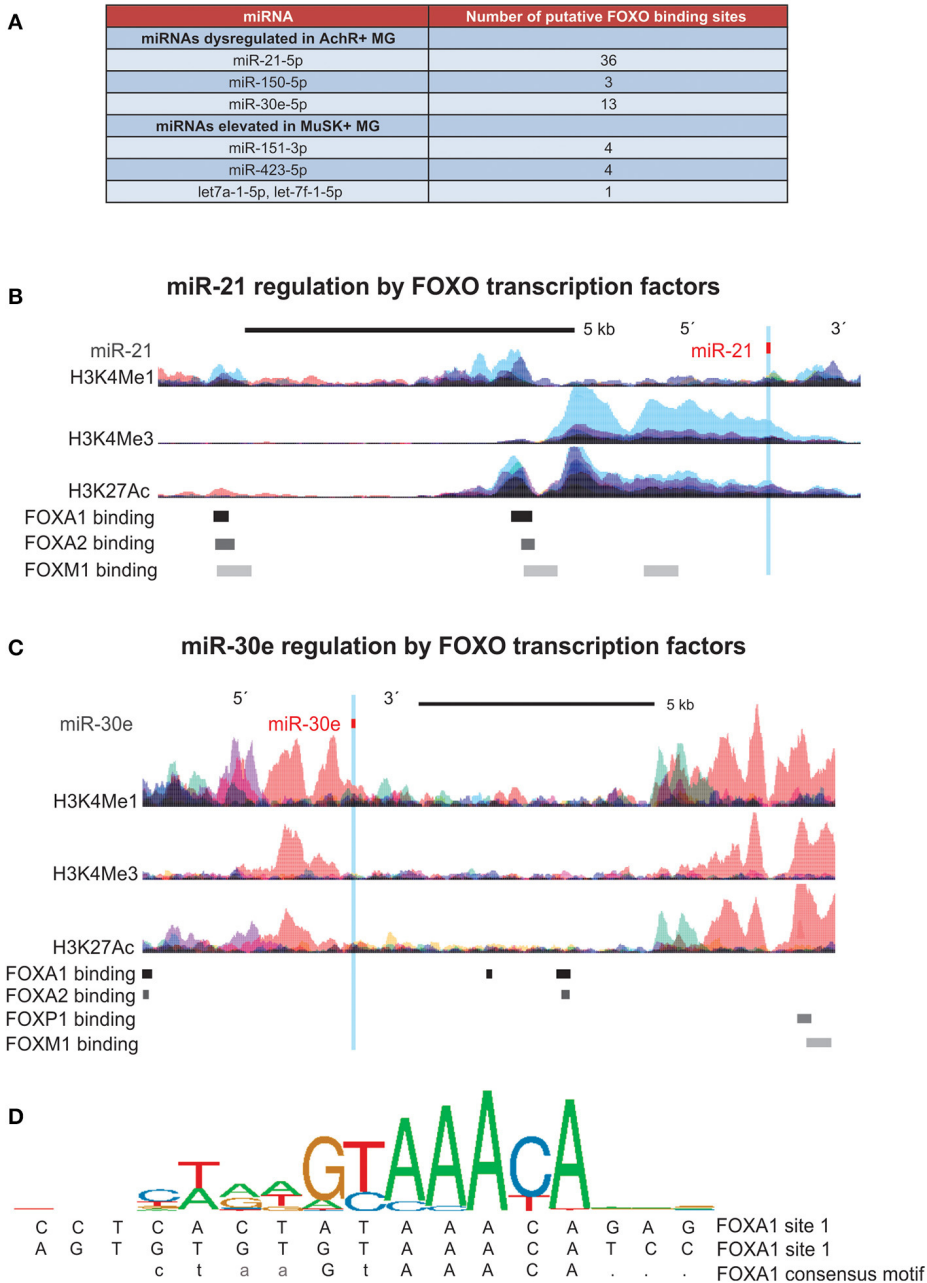
Promoter analysis of miRNAs indicates regulation by FOXO transcription factors. FOXO Transcription factor binding sites and histone (H3) modifications that mark regulatory regions were examined using ChIP-seq data from ENCODE. DNA binding motifs for each transcription factor were identified through the Factorbook repository. **(A)** Table of all FOXO binding site found in regulatory regions of miRNAs dysregulated in both AchR+ and MuSK+ MG, demonstrating a high number of binding sites within miRNAs specific to the AchR+ subtype. **(B,C)** Schematic illustrating binding sites for FOXO transcription factors within the **(B)** miR-21 locus (seven of 26 binding sites shown) and **(C)** miR-30e locus (seven of 13 binding sites shown). Corresponding epigenetic modification maps are provided showing the location of histone modifications associated with active promoters (H3K4me3) and poised/active enhancers (H3K4me1 and H3K27Ac) in the immediate vicinity of both loci. **(D)** Sequence logo pictogram of base frequency at FOXA1 binding sites, with the consensus FOXA1 motif provided immediately below. Also provided are two representative FOXA1 binding site sequences near miR-30e, listed in order from the 5′ to 3′ direction.

Due to the different signature of circulating miRNAs in AChR+ MG vs. MuSK+ MG, we next in silico determined the set of transcription factors that more specifically regulate the expression of these subtype specific miRNAs. AChR+ MG miRNAs showed a stronger overall regulation by the FOXO transcription factors, considering that miR-21-5p, miR-150-5p, and miR-30e-5p together possessed 52 binding sites for FOXO transcription factors (Figure 2A). On the contrary, MuSK+ miRNAs possessed less binding sites for the FOXO family (nine binding sites, Figure 2A) and instead had the most binding sites for the FOS, Jun, JunD, and Stat3 proteins. Further, in-depth analysis of each miRNA regulatory region revealed binding sites for the FOXO transcription factors within the miR-21 and the miR-30e loci (Figures 2B–D).

### Higher miR-21-5p Levels in Female Immunosuppression Naïve MG Patients

**Figure 3:**
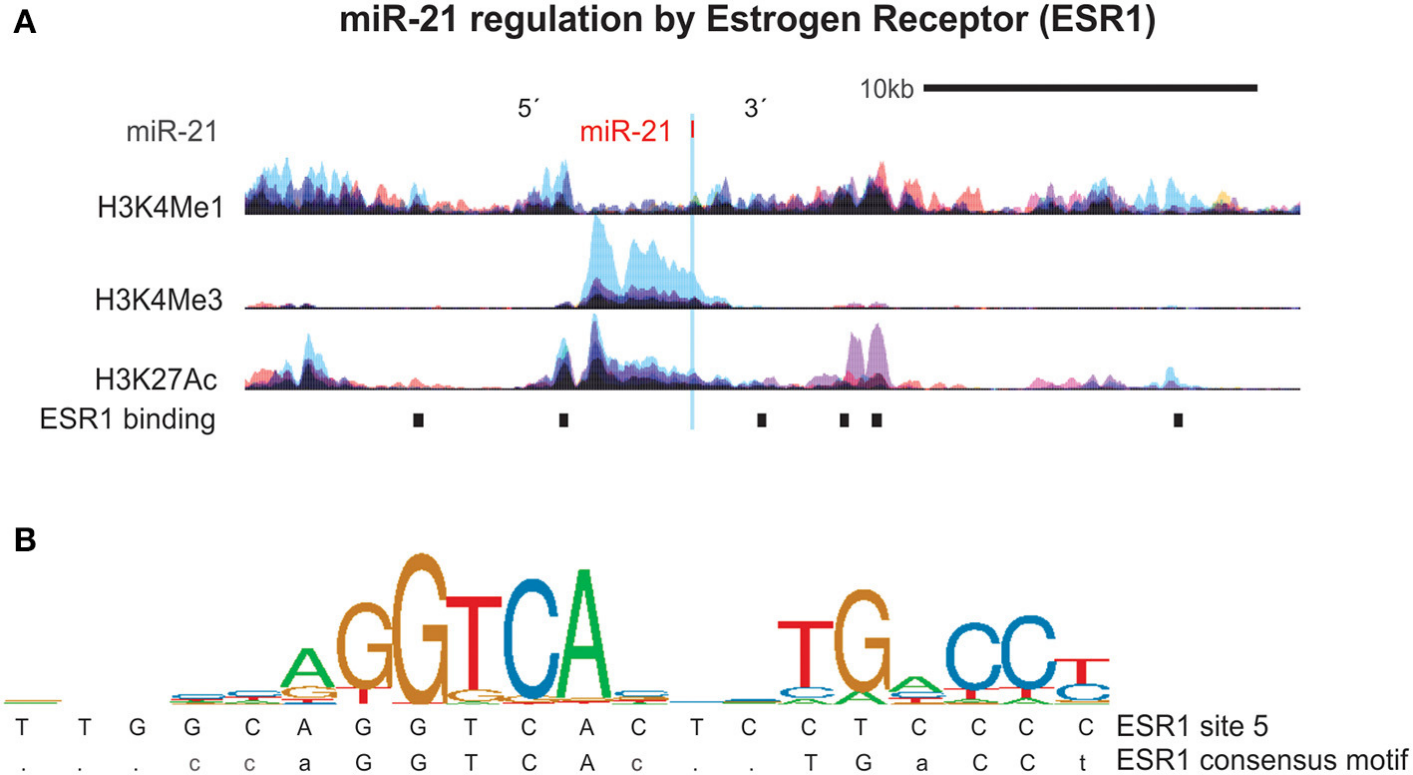
miR-21 is regulated by the Estrogen Receptor 1 (ESR1). **(A)** Estrogen receptor 1 (ESR1) binding sites and histone (H3) modifications that mark regulatory regions were examined using ChIP-seq data from ENCODE as detailed in Figure 2. Shown are the six ESR1 binding sites in the region surrounding the miR-21 DNA locus. **(B)** Sequence logo pictogram of base frequency at ESR1 binding sites, with the consensus ESR1 motif provided immediately below. Also provided is a representative ESR1 binding site sequences near miR-30e, listed in order from the 5′ to 3′ direction.

**Figure 4:**
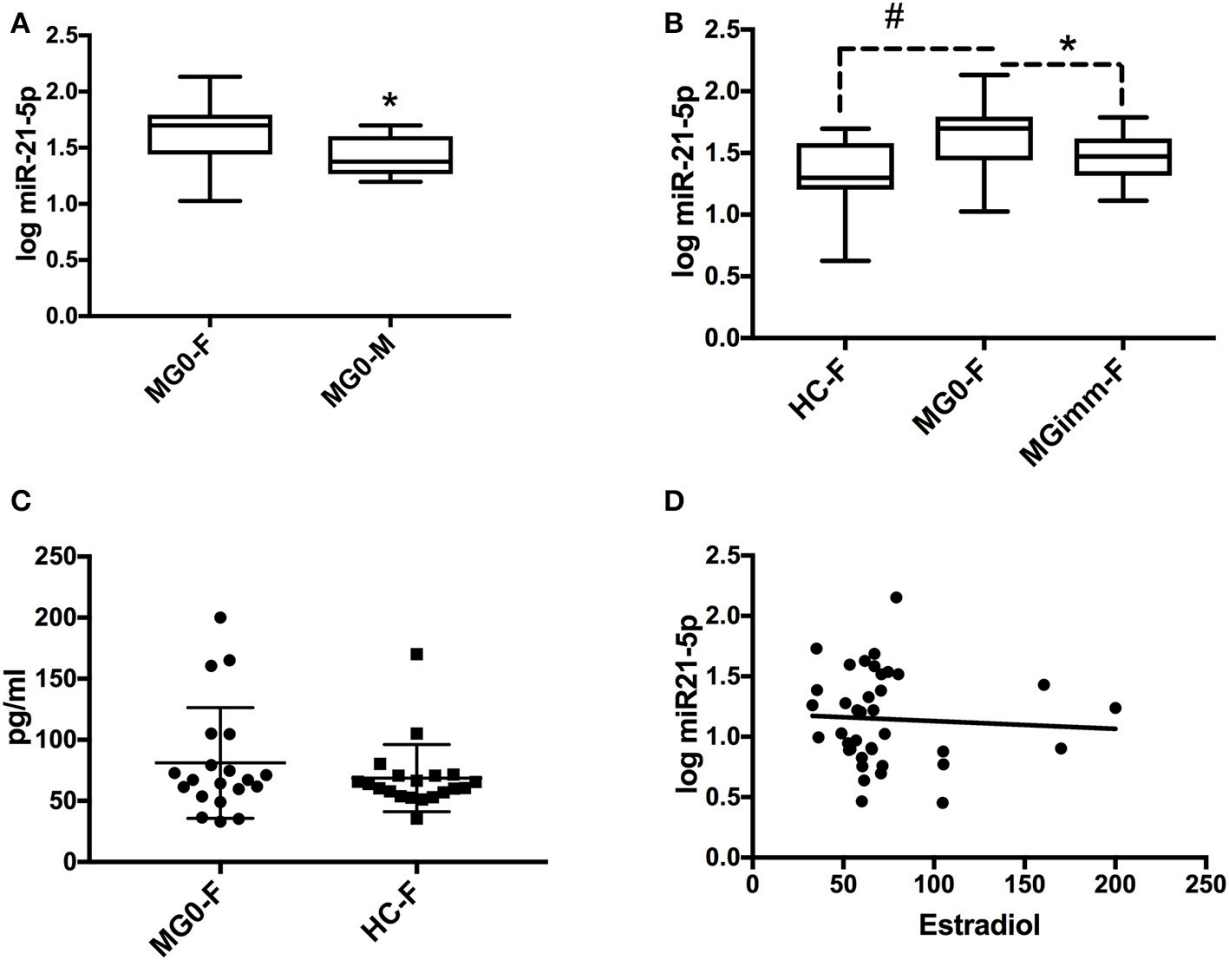
miR-21-5p levels are highest in female MG patients without immunosuppression. **(A)** miR-21-5p levels in female (MG0-F; *N* = 27) and male MG (MG0-M; *N* = 10) patients without immunosuppression. **(B)** miR-21-5p levels in female healthy controls (HC-F; *N* = 27), MG0-F and MG patients with immunosuppression (MGimm-F; *N* = 15). Data in panels **(A,B)** are shown in logarithmic (log) scale and the boxplot refers to mean ± SD and whiskers state min to max values. **(C)** Serum 17-ß-estradiol in female MG patients (*N* = 20) vs. female age matched healthy controls (*N* = 20). **(D)** Correlation between 17-ß-estradiol and miR-21-5p among female healthy controls (*N* = 20) and MG patients without immunosuppression (*N* = 20). Spearman *R* = −0.053; *p* = 0.75. *p < 0.05; ^#^*p* < 0.0001.

We next focused on the relationship of estrogen and miR-21-5p, as our bioinformatics analysis demonstrated heavy regulation of this miRNA by ESR1, showing six binding sites within its promoter/enhancer region (Figures 1, 3A,B). Based on this, we tested the hypothesis that miR-21-5p levels would be markedly different between male and female MG patients. To do this, we re-analyzed miR-21-5p levels from archival data of AChR+ MG patients and healthy controls, this time grouping them by gender (5). There was no difference between serum miR-21-5p levels of healthy female controls (*N* = 27) and healthy male controls (*N* = 27; *p* = 0.33) or between female (*N* = 27) or male (*N* = 10) MG patients with immunosuppression (*p* = 0.90). However, female MG patients without immunosuppression had significantly higher values than male MG patients without immunosuppression (*p* = 0.015; Figure 4A). miR-21-5p levels were also higher in female MG patients without immunosuppression compared to healthy female controls (*p* = 0.0001; Figure 4B) as well as female MG patients with immunosuppression (*p* = 0.019, Figure 4B). One-way ANOVA did not reveal any difference in miR-21-5p levels between the groups of AChR+ and AChR/MuSK- patients (without and with immunosuppression, respectively; data not shown; *p* = 0.21).

We next explored whether age could play a role for miR-21-5p levels. However, correlation analysis did not reveal any relationship between age and miR-21-5p levels in healthy female controls (*R* = 0.25; *p* = 0.21) nor in male healthy controls (*R* = −0.04; *p* = 0.85). Neither was there any correlation between miR-21-5p levels and age in female MG patients without immunosuppression (*R* = 0.17; *p* = 0.39). A significant age correlation was seen however for miR-21-5p in male MG patients without immunosuppression (*R* = 0.75; *p* = 0.017), i.e., increasing miR-21-5p levels with increasing age.

Based on the link between ESR1 and miR-21-5p, we assumed that serum estradiol levels could be correlated to miR-21-5p levels. Therefore, we next assessed estrogen levels in female controls vs. female MG patients. While there was no significant difference between 17-β-estradiol levels in female MG patients compared to their age-and sex matched controls, there was a trend toward increased serum estradiol in female MG patients (*p* = 0.07; Figure 4C). Nevertheless, there was no clear correlation between miR-21-5p and 17-β -estradiol levels when taking the entire female cohort, i.e., immunosuppression naïve MG patients and healthy controls, into consideration (*p* = 0.75; Spearman *R* = −0.05; Figure 4D). In summary, the observed higher miR-21-5p levels in female MG patients without immunosuppression compared to male patients does not appear to be mediated by estradiol levels alone.

Finally, in order to determine whether estradiol would induce expression of miR-21-5p in a human T cell line, these cells were treated with 17-β-estradiol at different concentrations and duration. Treatment with 17-β-estradiol for 24, 48, and 72 h did not result in a significant increase in miR-21-5p levels (data not shown). Further, treatment with the estrogen receptor inhibitor tamoxifen did not induce significant reduction of miR-21-5p levels (Supplementary Figure 1).

### NF-κB Pathway Up-Regulates Expression of MG-Associated miRNAs

**Figure 5:**
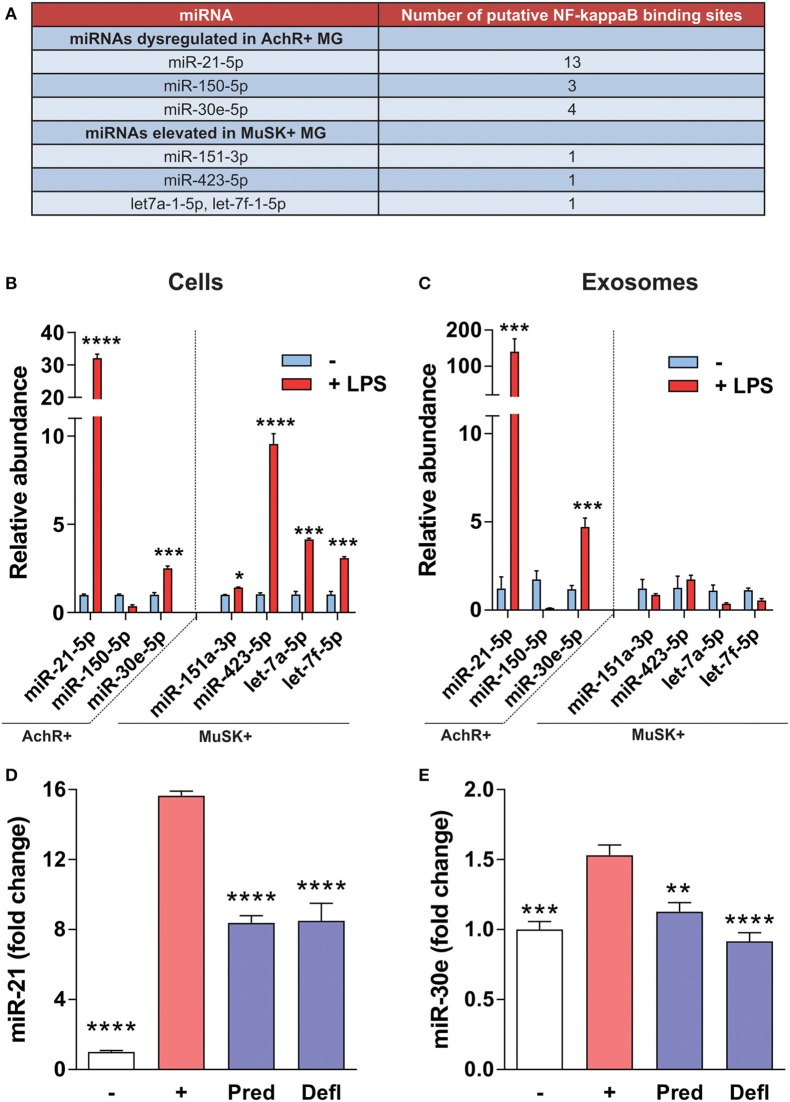
NF-κB-mediated regulation of MG miRNAs. **(A)** Table shows the number of putative NF-κB binding sites within the regulatory regions of each miRNA listed. **(B,C)** Inflammatory signaling was induced in RAW 264.7 for 48 h using LPS. Expression of miRNAs was assayed by qRT-PCR. For each figure LPS treatment was compared to the respective no treatment control. **(B)** In a murine macrophage cell line, LPS induces miR-21-5p, miR-30e-5p, miR-151a-3p, miR-423-5p, let-7a-5p, and let-7f-5p **(C)** LPS induces packaging of miR-21-5p and miR-30e, but not other MG-associated miRNAs, into exosomes (*n* = 4, student's two-tailed *t*-test, ****p < 0.0001; ***p < 0.001; *p < 0.05). **(D,E)** RAW 264.7 macrophages were pretreated with the indicated drug at 10 μM and inflammatory signaling was induced for 24 h using LPS. Expression of miRNAs within cells was assayed by qRT-PCR for **(D)** miR-21-5p and **(E)** miR-30e-5p, respectively (*n* = 4 ****p < 0.0001; ***p < 0.001; **p < 0.01, ANOVA with *post hoc* vs.; [–] = no LPS control, [+] = LPS plus vehicle, Defl, deflazacort; Pred, prednisolone.

Based on the finding that AChR+ MG-associated miRNAs have a large number of putative NF-κB binding sites within the regulatory domains (Figure 5A), the effect on the expression of these miRNAs was assayed through induction of inflammatory signaling in a murine macrophage cell line. Upon inflammatory induction by LPS, which activates NF-κB through TLRs, such as TLR4, levels of miR-21-5p, miR-30e-5p, miR-151a-3p, miR-423-5p, let-7a-5p, and let-7f-5p were significantly elevated (Figure 5B). In addition, packaging of miR-21-5p and miR-30e-5p into exosomes was induced by inflammation (Figure 5C). Cell-specific expression of miRNAs was then assessed after anti-inflammatory treatment. Macrophages pretreated with the immunosuppressive treatment prednisolone and deflazacort attenuated the induction of the same miRNAs, miR-21 and miR-30e (Figures 5D,E).

## Discussion

Research on circulating miRNAs in autoimmune disorders, including MG, has developed drastically in recent years. In this study, we examined the transcription factors that regulate MG-associated miRNAs in order to understand what drives the dysregulated serum miRNA profile observed in MG patients. Moving forward, understanding these mechanisms will have important implications toward immunosuppressive and anti-inflammatory drug development in MG. Pathway-based analyses that combine information across multiple genes into a limited number of molecular networks have been found to be a powerful approach that can also be used for miRNAs. The majority of identified transcription factors for MG-associated miRNAs are involved in autoimmunity-related pathways, especially the FOXO proteins, AP-1 transcription factors, NF-κB, IRF, and STAT family and NFAT. In line with this, the AChR+ MG-associated miRNAs might undergo stronger overall regulation by the FOXO transcription factors, considering the 52 binding sites in miR-21-5p, miR-150-5p, and miR-30e-5p for FOXO transcription factors. In AChR+ MG, T regulatory cells (Tregs) have reduced suppressive activity and one of the potential causes of this is decreased expression of FOXP3 in the MG thymus and peripheral blood (15, 29). The Treg specific transcription factor, Fork head/winged-helix transcription factor (FOXP3), has been shown to regulate both the development and the function of Tregs (30). FOXP3 expression is partly controlled by the phosphorylation of signal transducer and activator of transcription 5 (STAT5) induced by the IL-2 transduction pathway and low FOXP3 levels in Tregs may result from decreased STAT5 phosphorylation after IL2-signaling (31). The subtype of MuSK+ MG and AChR+ MG differ in several ways, mainly in that MuSK antibodies are of IgG4 subclass (32, 33), whereas the AChR antibodies are of IgG1 subclass. Further in MuSK+ MG, thymus pathology is normal compared to AChR+ MG (34), where thymus hyperplasia is considered to play a major role. In line with this, the circulating miRNA profile differs between these antibody subtypes of MG and thus it could be anticipated that the transcription factor profile is also different, as encountered here.

The proinflammatory NF-κB signaling pathway is related to inflammatory diseases and stimulated by TNF-α and IL-1. NF-κB had 23 binding sites across all MG-associated miRNAs, the majority being located in the AChR+ MG-associated miRNA genes. This is in support of a large study on almost 1,200 European MG patients with early onset AChR+ MG, where several risk genes were found related to the NF-κB signaling pathway (35). Here, the genetic associations to MG outside the HLA complex indicate VAV1, a key signal transducer crucial for T and B cell activation and BAFF, a cytokine important in the proliferation and differentiation of B cells. Combined VAV1 and BAFF haplotypes conferred a greater risk in combination and in addition to CD86, and these share the same signaling pathway, NF-κB (35). It was therefore not entirely surprising to find that NF-κB regulates transcription of the miRNAs found to be upregulated in AChR+ MG. Together, these data implicate that patients with early onset AChR+ MG may very well be predisposed to dysregulated proinflammatory signaling. miR-21-5p is also regulated by NF-κB, which would, in part, explain why MG patients on immunosuppressive drug therapy, i.e., mainly prednisone, have reduced levels of miR-21 compared to patients without immunosuppression (5). Nevertheless, other immunomodulatory medications such as azathioprine with a slower onset treatment effect and more long-acting properties, potentially have different effects on miR-21-5p and miR-150-5p as compared to the more rapid onset treatment effect of prednisone (36).

After querying ChIP-seq data to understand how MG-associated miRNAs are regulated, we functionally tested NF-κB-specfic miRNA regulation in a murine macrophage cell line and ESR1-specific miRNA regulation in a human T cell line. Our results have important implications about how the overexpression of MG-associated miRNAs may occur, but they also have their limitations. For instance, administration of LPS along with prednisone/deflazacort pre-treatment in murine macrophage cells serves to confirm that NF-κB can indeed induce expression of MG-associated miRNAs (miR-21-5p, miR-30e-5p, miR-151a-3p, miR-423-5p, let-7a-5p, and let-7f-5p) within cells and that blocking NF-κB can attenuate expression of a subset of these miRNAs (miR-21-5p and miR-30e-5p). However, in future studies it will be important to determine how these miRNAs respond to inflammation in individual patients that may have different levels and activation of NF-κB/NF-κB signaling. We also detected miR-21-5p and miR-30e in the exosomes of murine macrophages in response to LPS administration. This observation suggests that in MG patient serum, miR-21-5p and miR-30e may have originated from inflammatory cells that shed miRNA-containing exosomes in response to elevated inflammation. In future experiments it will be important to perform these experiments in patient-derived cell lines to determine (1) the totality of cell/tissues types that may be responsible for releasing miRNAs into the serum via exosomes and (2) other transcription factors might drive miRNA packaging into exosomes.

We analyzed miR-21-5p expression in a human T-cell line after treatment with estradiol, but did not, however, see significant induction. It is possible that estradiol was not sufficient to induce miR-21-5p expression because other transcription factors that co-regulate this miRNA (such as NF-κB) confounding the data. This is a likely scenario given that in female MG patients, we only observed significant elevation of serum miR-21-5p in when patients on immunopressive drugs were removed from analysis; this suggests heavy regulation of miR-21-5p by NF-κB. It is also possible that already high baseline ESR1 activation in this cell line prevented any additional activation of miR-21-5p upon estradiol administration.

To build our bioinformatics and cell line results, in future studies it will be important to determine how each of the identified transcription factors is expressed and how these collectively coordinate miRNA transcription in MG. While the cell culture experiments performed here were to determine if, in our hands, specific miRNAs are regulated by NF-κB and ESR1, these experiments do not yield the full picture of how these MG-associated miRNAs become dysregulated in MG. While the data presented here allow us to postulate on which transcription factors are responsible for driving the expression of AChR+ and MuSK+ MG-associated miRNAs, what would be quite interesting in future studies is to derive macrophage and T-cells from AChR+ and MuSK+ patients and perform similar treatments, as mentioned above. Further, we could perform RNA-seq in comparison to healthy controls to look at potential upregulation of specific transcription factors. In follow up studies, such experiments will help us to determine which transcription factors would be the best therapeutic targets for AChR+ and MuSK+ MG.

There are distinct sex-associated differences in the expression of tissue-specific antigens that are controlled by the autoimmune regulator, a key factor for central tolerance in the autoimmune response. A recent study showed that in females, estrogen induces epigenetic changes in the autoimmune regulator (AIRE) gene, causing reduced AIRE expression below a threshold that increases female susceptibility to autoimmune diseases and in particular MG (37). In addition, worsening in female MG patients have been described in particular phases of the normal menstrual cycle, commonly (up to 50%) with symptoms being at their worst just before the next menstrual flow (38), when both progesterone and estrogen levels are the lowest. Previous studies indicate that 17ß-estradiol participate in the tolerization process by decreasing the expression of α-AChR and HLA-DR proteins and increases the expression of type I interferon and related molecules in thymic epithelial cells (37). With all the aforementioned effects of 17-β-estradiol on different aspects in MG in mind, the finding that ESR1 could regulate miR-21-5p, let-7a, and let-7f through binding sites is intriguing. This is in line with the higher levels of miR-21-5p in female immunosuppressive naïve MG patients compared to their male counterparts. However, we did not find a direct correlation between serum miR-21-5p and estradiol levels in female healthy controls or female MG patients without immunosuppression, indicating that other factors may influence the measurable circulating levels. Further, since 17-β-estradiol in women fluctuates during different phases of the menstrual cycle (39) and we did not sample the women during only one phase so this makes the interpretation of 17-β-estradiol levels more difficult.

In conclusion, the findings of our study highlight the connection between the MG-associated miRNAs and signaling pathways that govern inflammatory events and risk factors known to be important at least in AChR+ MG. This supports the important role of having biomarkers, such as miRNAs, that also are connected to the autoimmune events underlying the disease.

## Data Availability Statement

The datasets generated for this study are available on request to the corresponding author.

## Ethics Statement

The studies involving human participants were reviewed and approved by the Ethical Review Board of Uppsala University (Dnr 2010/446). The patients/participants provided their written informed consent to participate in this study.

## Author Contributions

AF, CH, TP, and AP contributed conception and design of the study. AF, CH, Y-FH, and CT performed experiments. AF and AP performed statistical analysis. AP and AF wrote the first draft of the manuscript. CH, Y-FH, and TP wrote sections of the manuscript. All authors contributed to manuscript revision, read and approved the submitted version.

### Conflict of Interest

The authors declare that the research was conducted in the absence of any commercial or financial relationships that could be construed as a potential conflict of interest.

## Supplementary Material

Supplementary Figure 1 Human T-cell line KE-37 was treated for 24, 48, and 72 h, respectively, with ß-estradiol at 0 nM (control group), 5 and 50 nM. The relative expression of miR-21-5p (%) compared to the control group is displayed on the y-axis.
