# Supplementary figures and images for: Estrogen Receptor, Inflammatory, and FOXO Transcription Factors Regulate Expression of Myasthenia Gravis-Associated Circulating microRNAs
Source: Front Immunol. 2020 Feb 21;11:151. doi: 10.3389/fimmu.2020.00151 (PMC7046803; doi:10.3389/fimmu.2020.00151)

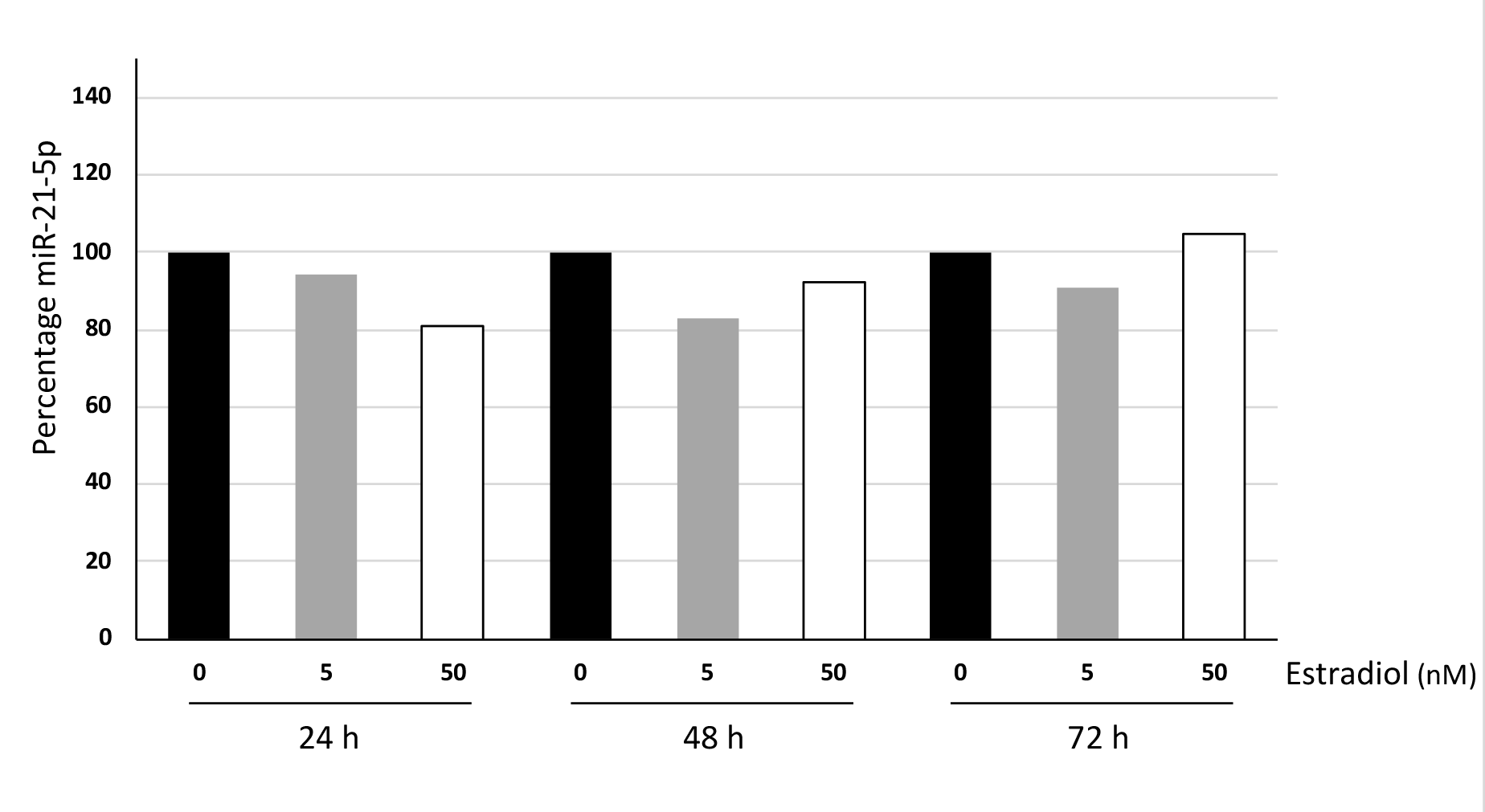

Supplement: Supplementary Figure 1 — Human T-cell line KE-37 was treated for 24, 48, and 72 h, respectively, with ß-estradiol at 0 nM (control group), 5 and 50 nM. The relative expression of miR-21-5p (%) compared to the control group is displayed on the y-axis. [file Image_1.tif]
